# Cluster-Based Ensemble Learning Model for Aortic Dissection Screening

**DOI:** 10.3390/ijerph19095657

**Published:** 2022-05-06

**Authors:** Yan Gao, Min Wang, Guogang Zhang, Lingjun Zhou, Jingming Luo, Lijue Liu

**Affiliations:** 1 School of Automation, Central South University, Changsha 410083, China; gaoyan@csu.edu.cn (Y.G.); 214611109@csu.edu.cn (M.W.); csu_0918@163.com (L.Z.); 2 Xiangya School of Medicine, Central South University, Changsha 410083, China; zhangguogang@csu.edu.cn (G.Z.); jmluo0618@139.com (J.L.)

**Keywords:** aortic dissection, imbalanced data, screening, clustering, bagging

## Abstract

Aortic dissection (AD) is a rare and high-risk cardiovascular disease with high mortality. Due to its complex and changeable clinical manifestations, it is easily missed or misdiagnosed. In this paper, we proposed an ensemble learning model based on clustering: Cluster Random under-sampling Smote–Tomek Bagging (CRST-Bagging) to help clinicians screen for AD patients in the early phase to save their lives. In this model, we propose the CRST method, which combines the advantages of Kmeans++ and the Smote–Tomek sampling method, to overcome an extremely imbalanced AD dataset. Then we used the Bagging algorithm to predict the AD patients. We collected AD patients’ and other cardiovascular patients’ routine examination data from Xiangya Hospital to build the AD dataset. The effectiveness of the CRST method in resampling was verified by experiments on the original AD dataset. Our model was compared with RUSBoost and SMOTEBagging on the original dataset and a test dataset. The results show that our model performed better. On the test dataset, our model’s precision and recall rates were 83.6% and 80.7%, respectively. Our model’s F1-score was 82.1%, which is 4.8% and 1.6% higher than that of RUSBoost and SMOTEBagging, which demonstrates our model’s effectiveness in AD screening.

## 1. Introduction

Aortic dissection (AD) is a medial rupture caused by intramural hemorrhage, which leads to the separation of the aortic wall layer, followed by the separation of the true and false lumen [1]. AD is a dangerous cardiovascular disease with many complications and high mortality. Mortality can reach as high as 50% within 48 h of onset and 60–70% within a week [2,3]. Rapid diagnosis is very important for the treatment of AD.

However, the clinical manifestations of AD are complex and changeable. AD patients often lack specific symptoms and signs. Additionally, the location, lesion degree and scale of AD are different. Clinicians tend to observe the common symptoms of AD to diagnose it, such as chest pain and back pain. However, for patients without pain, atypical symptoms make the diagnosis more difficult. Thus, AD is easily missed or misdiagnosed [4]. More than 1/3 of AD cases are missed in actual cases of AD [5,6,7], and the rate at which acute aortic syndrome is missed in the emergency room is close to 80% [8]. The rarity of AD is also one of the reasons for the high rate of missed diagnosis. The incidence of AD is about 11.9 cases per 100,000 people [9], and the incidence of AD in the emergency room is 5.93–24.92 cases per 100,000 people [10]. With the popularization of imaging technologies, such as computerized tomography angiography (CTA) and magnetic resonance imaging (MRI), the diagnosis rate of AD has increased significantly [4].

In Chinese rural and remote areas, many hospitals lack medical imaging equipment. However, routine examinations are common in every hospital. Because of AD’s rarity, many clinicians in Chinese rural or remote hospitals have less experience diagnosing AD patients. Developing an AD screening model based on patients’ routine examination data could be an effective way to help clinicians identify patients at high risk of AD in the early phase and save patients’ lives.

With the rapid development of artificial intelligence, machine learning methods were adopted in the medical domain [11,12,13]. Our AD screening model based on machine learning includes two parts: the construction of an AD dataset and the application of a supervised learning algorithm to build a classifier for screening AD patients. 

In some disease screening models, the combination of multiple signs and symptoms promises to increase diagnostic accuracy [14,15]. The screening model for AD disease is the same. Due to AD disease having no specific signs and symptoms, we collected the patients’ symptoms, routine examination data, lifestyle habits and family genetic history from electrical health records in XiangYa Hospital to construct an AD dataset. T-SNE [16] was used to visualize the distribution of the AD data. 

Because of AD’s rarity, the ratio of non-AD patients to AD patients in the AD dataset was extremely high. In order to solve the problem caused by data imbalance, we developed a cluster-based ensemble learning model: Cluster Random Undersampling Smote–Tomek Bagging (CRST-Bagging) to help clinicians screen for AD patients in clinical practice. In this model, we proposed a new resampling method: CRST, which can increase the number of AD patients by oversampling algorithm Smote and decrease the number of non-AD patients by kmeans++ and undersampling algorithm Tomek-link. It reduces the imbalance between AD patients and non-AD patients, which can help improve the accuracy of the screening model. In order to demonstrate the effectiveness of the CRST-Bagging model, we compared it with other classic imbalance methods on the AD dataset. Experimental results show that the proposed model is more effective than other models, which proves the effectiveness of our model.

The main contributions of this paper are as follows:

- We constructed an AD dataset based on patients’ routine examination data, lifestyle habits and family genetic history from EMRs from Xiangya Hospital. Data preprocessing and data visualization in the AD dataset were used to obtain prior knowledge of the AD data distribution to help us understand the data.
- An integrated sampling method—CRST—was proposed to reduce the imbalance ratio of the AD data effectively. CRST combines the advantages of kmeans++ and the Smote–Tomek algorithm. This method not only makes the collected non-AD patients and AD patients more representative but also ensures the randomness of sampling. CRST is suitable for dealing with a highly imbalanced dataset.
- The CRST-Bagging model was developed to help clinicians screen for AD patients. In the model, our proposed resampling method CRST and Bagging ensemble algorithm are combined to improve the robustness and generalization ability for AD screening.

The rest of this article is arranged as follows. In the second section, we introduced the related work. In the third section, we introduced the dataset, our CRST resampling method and the ensemble model CRST-Bagging for AD screening. In the fourth section, we presented our experimental results and evaluated the model’s performance. In the fifth section, we discussed our results and future work. Finally, the last section concludes the paper.

## 2. Related Work

With the accumulation of a large amount of medical data, researchers focused on using machine learning methods to help clinicians predict AD diseases based on image data. The CNN algorithm [17] can diagnose AD through plain CT image data and has achieved good results in accuracy, sensitivity and specificity. The application of this algorithm improves the diagnosis rate of AD patients who have atypical symptoms detected by conventional CT plain scan. Harris et al. [18] developed a CNN model based on enhanced CT image data, which can diagnose and classify AD and aortic rupture. The model recognizes the severity of the patient’s condition, so critically ill AD patients can be diagnosed first and obtain medical assistance. Cheng et al. [19] used a U-Net framework to classify AD based on contrast-enhanced CT images. Among 1000 CT images from 20 patients, the accuracy rate reached 85.0%. 

However, in actual situations, due to the lack of clinician experience or unsupported examination equipment, it is often difficult to carry out necessary imaging in time. This results in missing or misdiagnosing AD patients, which threatens patients’ lives. Therefore, researchers have worked to develop methods to screen AD patients in the early phase of their routine examination. Based on routine examination data, Huo et al. [20] applied many machine learning algorithms, including the Bayesian network, Naive Bayes, decision tree J48 and SVM, to classify AD patients in the emergency room. Their dataset is small, only containing 492 samples: 330 patients with AD and 162 patients misdiagnosed as AD, but the goal of their study was to decrease the number of misdiagnosed non-AD patients. Different from their work, our research goal is to screen for patients at high risk of AD. 

Applying a machine-learning algorithm to screen for AD disease has some problems. The rarity of AD leads to a serious imbalance in the dataset. If the traditional machine learning algorithm were applied to the AD dataset directly, the model would tend to be more biased towards the majority class. This causes a high missed diagnosis ratio. The resampling method is one of the most effective methods to solve imbalance problems. SMOTE [21,22] and Tomek-links [23,24] are excellent methods for oversampling and undersampling, respectively. However, single up-sampling or undersampling cannot deal with our extreme imbalance of large-scale AD datasets. Khushi et al. [25] studied the problem of data imbalance on two medical datasets related to lung cancer (the imbalance ratio is 24.7 and 25.2, respectively). Twenty-three class imbalance methods were compared. The results show that the Smote–Tomek method achieved the best results because the integrated sampling method has more advantages in highly imbalanced data. 

Due to the lack of special symptoms and signs of AD, it is very difficult to distinguish between AD and other cardiovascular diseases patients. This also makes it difficult for a machine-learning algorithm to determine the complicated boundaries between AD patients and non-AD patients. Algorithms combining the resampling method with the ensemble algorithm, such as RUSBoost [26] and SMOTEBagging [27], were used to improve the prediction ability when using an imbalanced dataset. The SMOTEBagging algorithm was applied in screening for AD patients. Liu et al. [28] investigated the performance of several different machine learning algorithms in the screening of AD patients based on routine examination data. The SMOTEBagging algorithm performed the best in their study. 

The works mentioned above provide us with ideas to develop a cluster-based ensemble learning model for aortic dissection screening. A more detailed explanation is given in Section 3.

## 3. Materials and Methods

**Figure 1:**
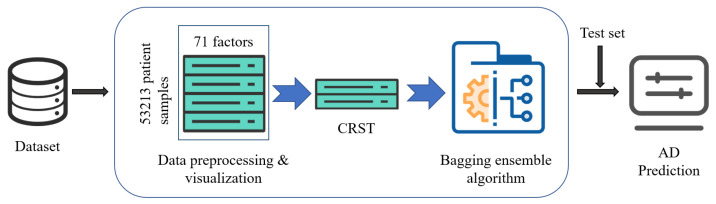
The structure of the CRST-Bagging model for AD screening.

The structure of the CRST-Bagging model for AD screening is shown in Figure 1. Data preprocessing (including missing value handling and data normalization) is carried out first. Cluster Random Undersampling Smote–Tomek (CRST) is proposed to resample the imbalanced dataset to reduce the imbalance ratio of the data. Finally, the Bagging ensemble model (CRST-Bagging) is used to construct a powerful classifier to predict AD. The methods are described in detail below. 

### 3.1. Dataset

#### 3.1.1. Data Overview and Visualization

Our AD dataset was created from the examination indicators of 53,213 patients, which were collected from Xiangya Hospital in Hunan Province from 2008 to 2016. The data include 802 patients with AD and 52,411 patients with other cardiovascular diseases (including viral myocarditis, myocardial infarction and coronary heart disease). The dataset has 71 features. These features were collected from electrical medical records and include the patients’ routine examination data, living habits and family genetic history. (see Table S1 of the Supplementary Materials). The AD patients’ ages range from 18 to 89 years. The average age of the AD patients in the dataset is 56. There are 206 AD patients with chest pain. The dataset has a high imbalance ratio, with approximately 67 times more non-AD samples than AD samples.

We also used a test set to verify our model’s classification performance and generalization ability better. The test set includes the examination indicators of 235 patients from the same hospital, of which there were 83 patients with AD and 152 patients with other cardiovascular diseases; the data format is the same as the aforementioned dataset. The AD patients’ ages range from 18 to 83 years. The average age of AD patients in the test set is 58. There are 56 AD patients with chest pain.

All the AD patients in these two datasets were diagnosed by CT, MRI, CTA or aortic surgery.

**Figure 2:**
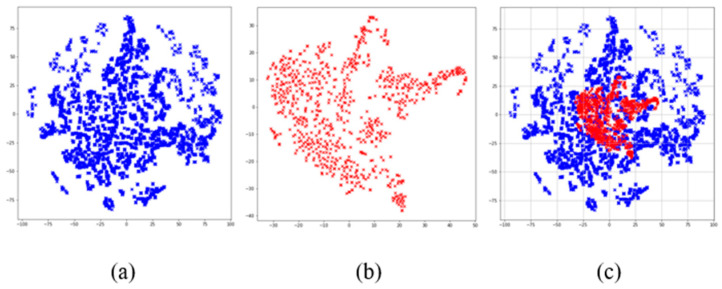
AD dataset t−SNE dimensionality reduction map. (**a**) The data distribution of non−AD patients after dimensionality reduction. (**b**) Data distribution of AD patients after dimensionality reduction. (**c**) The data distribution of non−AD and AD samples in the same space after dimensionality reduction.

In addition, we used the t-SNE algorithm to reduce the dimensions of the dataset, which is convenient for visualizing the data. The visualization results after dimensionality reduction are shown in Figure 2, where it can be seen that the data distribution of non-AD patients is agglomerated into many groups. This shows there is a certain similarity between some cases of non-AD patients. There is an obvious overlap between the two kinds of samples in space from the visualization of data distribution. This indicates that the boundary between AD and non-AD patients is ambiguous. Therefore, it is necessary to construct an ensemble classification model.

#### 3.1.2. Data Preprocess

In the AD dataset, there are some non-numerical features. The features such as smoking, drinking and family inheritance are binary coded. At the same time, the years of smoking and drinking are coded according to different degrees and ranges. All the numeric data are scaled to [0, 1].

**Figure 3:**
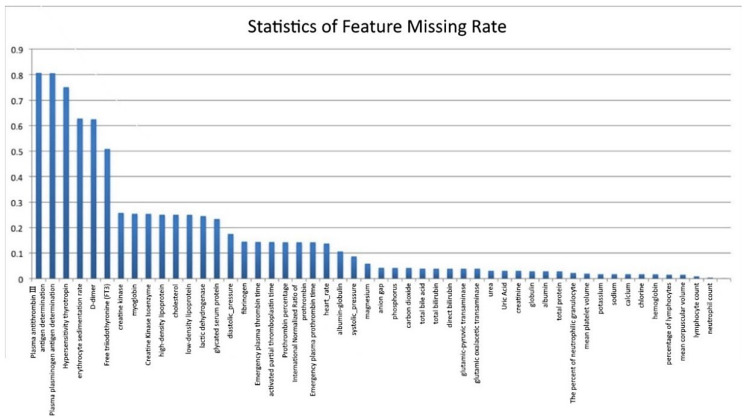
Feature Missing Rate Statistics.

We compiled statistics on the missing rate of samples and features in the original AD dataset, as shown in Figure 3 (the abscissa represents the features, and the ordinate represents the missing rate). Six features with a deletion rate of more than 50% were found, namely Antithrombin III antigen (AT: Ag) (missing rate is 81.5%), Plasminogen antigen (PLGAg) (missing rate is 80.7%), Hypersensitivity thyrotropin (S-TSH) (missing rate is 75.6%), erythrocyte sedimentation rate (ESR) (missing rate is 63.8%), D-dimer (missing rate is 62.6%) and free triiodothyronine (FT3) (missing rate is 51.9%). 

**Figure 4:**
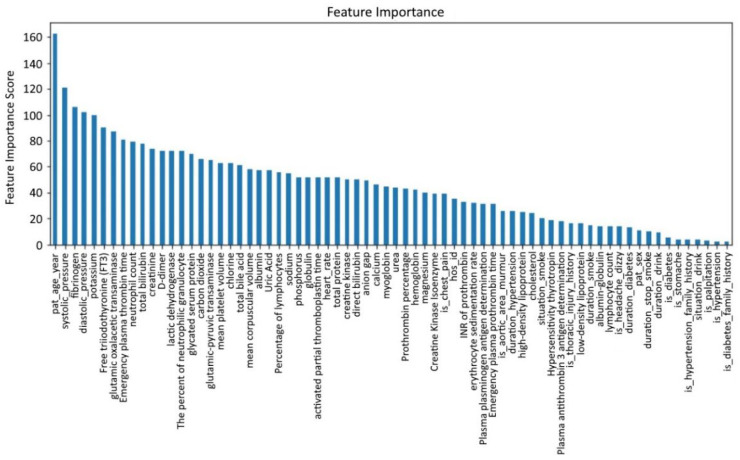
Feature importance analysis.

Due to the high missing rate of the aforementioned six features, it is difficult to fill them. The general method is to delete them. However, existing medical studies have shown that D-dimer is an important feature for the clinical diagnosis of AD, so it cannot be deleted directly. Although no research has proved that the other five features play a key role in the diagnosis of AD, direct deletion causes information loss. Therefore, the XGBoost method is used to analyze feature importance [29,30]. The result is shown in Figure 4, with the abscissa as a feature and the ordinate as feature importance scores.

From Figure 4, we find that among the six features with a deletion rate greater than 50%, the feature importance scores of FT3 and D-dimer rank in the top 10, which indicates these two features are important for detecting whether a patient suffers from AD. Therefore, we only remove the four features: AT: Ag, PLGAg, S-TSH, ESR. Therefore, FT3 and D-dimer remain and are filled with features of the complete samples. The adjusted new dataset size is (5,321,367). 

In this paper, the missing value was filled by the method of random filling by class. Compared with ordinary random filling, the method of random filling by class fills the positive and negative samples. The missing values of the samples were randomly filled with the non-null values of the same kind of samples. This filling method can effectively avoid the intersection of feature values.

### 3.2. Cluster Random Undersampling Smote–Tomek Approach (CRST)

Figure 2 shows there are many small groups in the non-AD patients. According to the characteristic of the AD set, we used the Cluster Random Undersampling Smote–Tomek Approach (CRST). It combines the advantages of K-means++ and the Smote–Tomek sampling method.

First, the training samples in the majority class were clustered by the K-means++ algorithm, in which K was obtained by super-parameter optimization. Then random undersampling was carried out for each cluster. The degree of sampling p% can be determined by the actual situation. After the undersampling, the Smote–Tomek combined sampling method was used to form a new balanced dataset. By iterating these operations many times, we obtained several new balanced sub-datasets.

**Figure 5:**
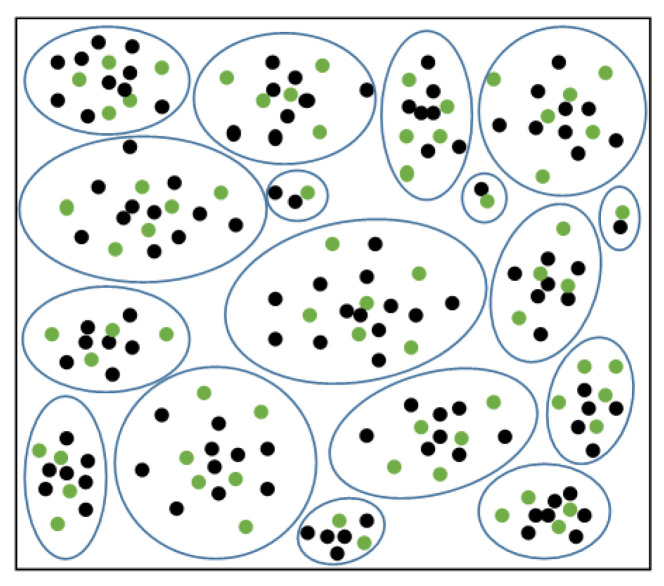
Schematic diagram of the undersampling process of majority samples.

The clustering of samples in the majority of classes is visualized in Figure 5. The green dots are the selected, remaining majority class sample points after p% random undersampling for each cluster. The sample points can be uniformly sampled in each cluster by undersampling, maintaining the original data distribution. 

**Figure 6:**
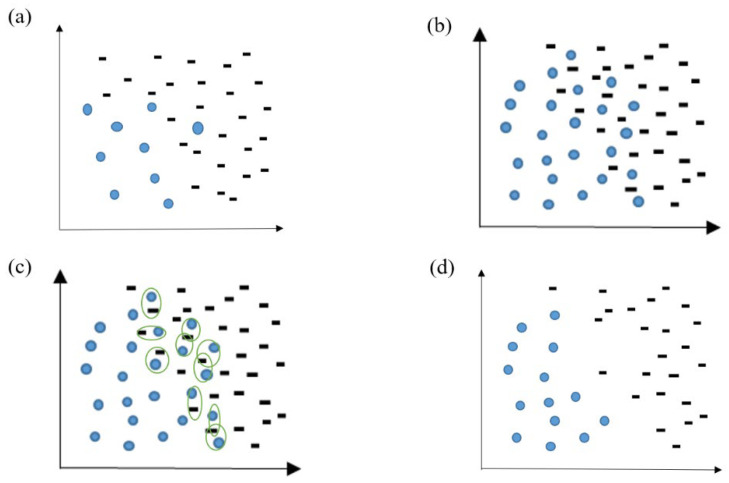
Raw dataset (**a**); dataset after SMOTE oversampling (**b**); Tomek-link recognition process (**c**); dataset after the boundary and noise samples are removed (**d**).

Finally, the Smote–Tomek(S-T) sampling method was applied to generate some minority samples. The sample loss caused by the undersampling was compensated, and the imbalance ratio was alleviated. As shown in Figure 6, S-T generated minority samples through the SMOTE method, while the Tomek-link method was adopted to solve the problem of fuzzy boundaries caused by the excessive generation of minority samples. This method can reduce the redundancy of samples. The algorithm’s procedure is shown in Table 1.

### 3.3. Ensemble Model Based on CRST

Due to the high-dimensional and complex characteristics of the AD dataset, it is necessary to construct a nonlinear classification model with strong generalization ability. On the basis of the CRST sampling method proposed in Section 3.2, combined with the Bagging [31] algorithm, a cluster-based ensemble model, CRST-Bagging, was developed to screen for AD patients. It overcomes the limitations of a single classifier.

**Figure 7:**
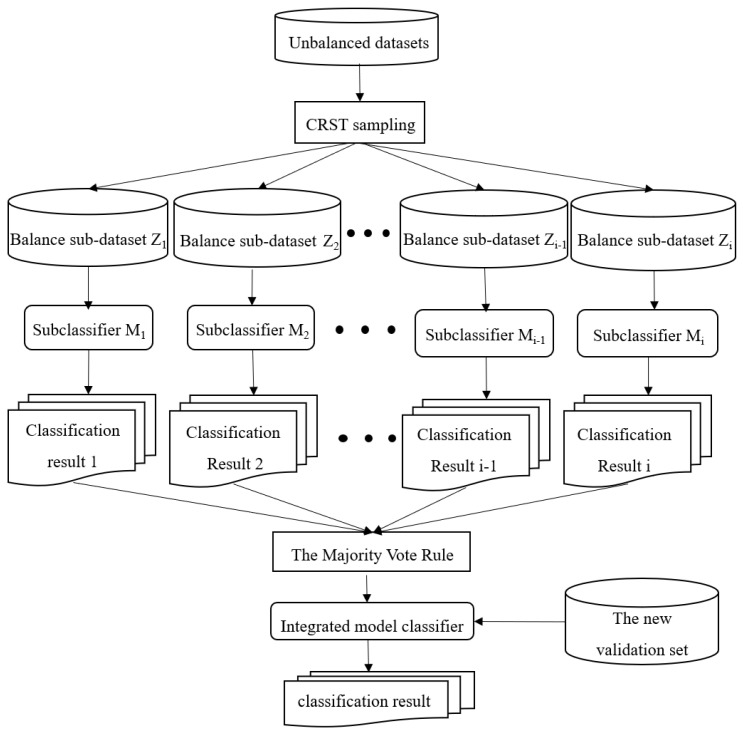
CRST-Bagging algorithm structure diagram.

The CRST-Bagging algorithm generates a new sample set $B=\left\{Z_{1},Z_{2},\cdots ,Z_{T}\right\}$ by using the CRST sampling method iteratively. Then each sub-sample set $Z_{i}$ is used to construct a sub-classifier $M_{i}$ separately. A complete ensemble model classifier can be obtained by integrating the results of the T sub-classifier. The integration rule used in the algorithm is the Majority Vote rule [32]. For the classifier, if $P_{i1}$ is greater than or equal to $P_{i2}$, then $R_{1}$ obtains one vote; if $P_{i1}$ is less than $P_{i2}$, then $R_{2}$ obtains one vote. $R_{1}$ and $R_{2}$ represent the sample category. This rule can be expressed by Formulas (1) and (2). The model structure is shown in Figure 7.

(1) $$R_{1}=\sum_{i=1}^{T}f\left(P_{i1},P_{i2}\right),\;R_{2}=\sum_{i=1}^{T}f\left(P_{i2},P_{i1}\right),\;where\;\;f\left(x,y\right)=\{\begin{array}{c} 0,\;x<y\\ 1,\;x>y \end{array}$$

(2) $$C=\begin{array}{c} argmax\\ j \end{array}R_{j}$$

## 4. Experiment

In order to evaluate the CRST sampling method’s effectiveness in resampling the AD dataset, we compared the method with the classical sampling methods Smote [21] and Smote–Tomek [23]. We also compared the classification performance of the CRST-Bagging model, RUSBoost [26] and SMOTEBagging [27] on the AD dataset. RUSBoost and SMOTEBagging are classic ensemble learning algorithms for imbalanced datasets.

### 4.1. Evaluation Metrics

Because of the extremely high imbalance ratio in the AD dataset, we use Precision, Recall and F1-score metrics to evaluate the CRST sampling method, CRST-Bagging model and other models. The formulas of these evaluation metrics are as follows:

(3) $$\mathrm{Recall}=\frac{\mathrm{TP}}{\mathrm{TP}+\mathrm{FN}}.$$

(4) $$\mathrm{Precision}=\frac{\mathrm{TP}}{\mathrm{TP}+\mathrm{FP}}.$$

(5) $$F1=\frac{2\times \mathrm{Precison}\times \mathrm{Recall}}{\mathrm{Precision}+\mathrm{Recall}}.$$

Here, TP is the number of true positives, FP is the number of false positives, TN is the number of true negatives and FP is the number of false negatives. The positive class indicates “AD patient”: the negative class indicates “non-AD patient”.

### 4.2. Validity Experiment of the CRST Sampling Method

We compared XGBoost, Smote, Smote–Tomek and CCST with the CRST sampling method on the AD dataset and used the seven-fold cross-validation to measure the sampling performance. In order to ensure the consistency of the experiment, XGBoost was used as the classifier for the comparative experiment. The parameters of the three methods were set as follows:

- Smote and Smote–Tomek(S-T): The samples in the majority class were randomly sampled, so the ratio of majority to a minority was 2:1. Then Smote and S-T were carried out so that the ratio of positive and negative samples after resampling was 1:1. There were 1604 samples in each category;
- CCST: The CCST method refers to the clustering of most class training sample sets by the K-means++ algorithm. N sample points closest to the center of each cluster were selected. Then the Smote–Tomek method was applied to balance the data. We set K = 802; N = 2. After undersampling, there were 1604 majority samples;
- CRST: The number of clusters K was obtained by the super-parameter selection, which was 30. First, 3.1% of each cluster class was selected for undersampling, then S-T was applied to the samples in the minority class. There were 1624 samples in each category.

The results are as follows:

The experimental results of the seven-fold cross-validation on the original dataset (5,321,367) are shown in Table 2. Our proposed resampling method CRST performed well, with the best value in recall (0.774) and F1-score (0.778), although S-T has the highest value in precision. The overall performance of CRST is the best. The performance of XGBoost is the worst among these algorithms because of the imbalanced AD dataset.

### 4.3. CRST-Bagging Model Effect Comparison Experiment

We also conducted experiments on the AD dataset to compare the prediction ability of CRST-Bagging, RUSBoost and SMOTEBagging. The experimental details of the various algorithms of this experiment are as follows:

- RUSBoost: The base learner type was decision tree C4.5, the number was 100 and the depth was 5;
- SMOTEBagging [28]: The number of clusters was set at K = 5, the base learner was decision tree C4.5, the number was 100 and the depth was 6;
- CRST-Bagging: The number of clusters was set at K = 50; p% = 3.1%;

Table 3 shows the experimental results of RUSBoost, SMOTEBagging and CRST-Bagging on the original dataset (53,213, 67) using seven-fold cross-validation. The results on the original dataset show CRST-Bagging performed best out of the three models. The precision, recall, and F1 score are 84.1%, 78.3%, and 81.1%, respectively. SMOTEBagging is the second-best, and RUSBoost is the worst.

In order to test the generalization ability of the model, we evaluated the models’ prediction ability on the test set (235, 67). The experimental results of the three algorithms are shown in Table 4. SMOTEBagging has the best precision on the test dataset (84.2%), but CRST-Bagging has the best recall value (80.7%) and the best F1 score (82.1%). CRST-Bagging has the best overall performance.

Table 5 shows the confusion matrix of the three algorithms on the test dataset. SMOTEBagging has one less misdiagnosis case than CRST-Bagging, but CRST-Bagging has three fewer missed diagnosis cases than SMOTEBagging. RUSBoost performed the worst.

**Figure 8:**
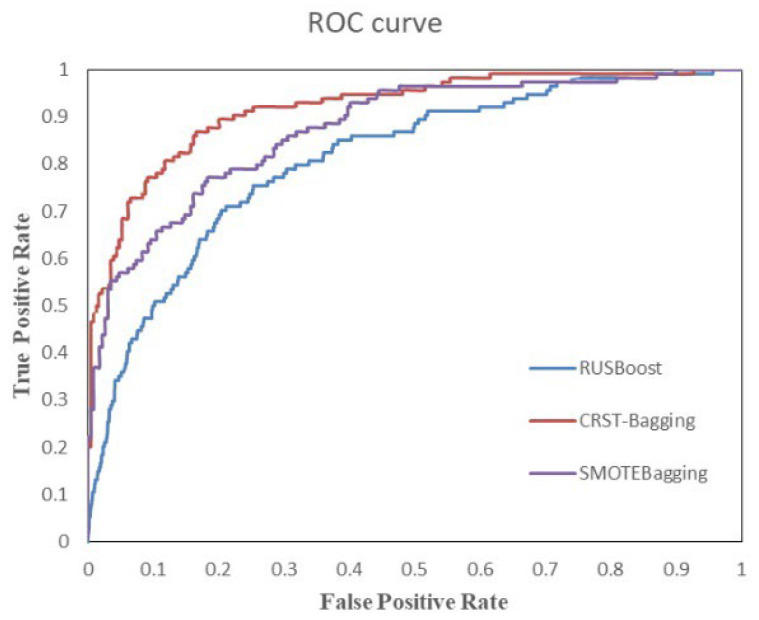
ROC graph of RUSBoost, SMOTEBagging, CRST-Bagging on the test set.

Figure 8 shows the ROC curve of three algorithms on the test set. It reflects the relationship between classification specificity and sensitivity. The area under the curve (AUC) represents the diagnostic effect. The larger the AUC value, the better the diagnostic effect of the model. The ROC plot in Figure 8 also demonstrates that the CRST-Bagging works best, which indicates that the model is better at predicting patients with AD.

## 5. Discussion

AD is a rare and high-risk cardiovascular disease. Its complex clinical manifestations and various atypical symptoms lead to serious misdiagnosis and missed diagnosis. In many Chinese basic hospitals, where the equipment is not advanced enough, it is difficult for patients to perform the examination items such as CT and MRI. AD screening is an effective way to determine which patients need further examination. Our study uses routine examinations and machine learning to build a screening model for AD in its early phase.

Because of the rarity of AD, the imbalance of our AD dataset is extremely high. We developed a CRST resampling method to solve the imbalance problem. Compared to XGBoost, the performance of XGBoost was the worst, which means that the extreme imbalance between AD patients and non-AD patients leads the bad diagnostic ability. Compared to other classic resampling methods, CRST performed best overall, with the highest values in recall (77.4%) and F1 (77.8%). This is because two undersamplings (Kmeans++ and Tomek) and one oversampling (Smote) are carried out in CRST, which can effectively reduce the imbalance in the AD dataset. In CRST, Kmeans++ is used to cluster non-AD patients, and a certain percentage of non-AD patients is randomly selected from the clusters. It not only makes the selected non-AD patients effectively represent the characteristics of most non-AD patients but also ensures the randomness of sampling. CRST can relieve the obstacles that imbalanced data bring to the construction of AD screening models.

We compared the CRST-Bagging model with RUSBoost and SMOTEBagging. The experimental results on the original dataset (5,321,367) indicate that the CRST-Bagging model performed the best among the three models (Table 3). The experiment on the test dataset is to examine the generalization ability of the model. The results (Table 4) show that CRST-bagging achieved the best overall performance: best recall value (78.3%) and best F1 value (81.1%). The confusion matrices of the three models are listed in Table 5. The CRST-Bagging missed 16 diagnoses, less than RUSBoost [23] and SMOTEBagging [24]. There are 13 misdiagnosed cases, only one case more than SMOTEBagging. This indicates that CRST-Bagging can effectively decrease the number of missed cases without increasing the number of misdiagnosis cases substantially. Because AD is a serious acute disease with high mortality, missed diagnosis is more dangerous than a misdiagnosis.

Clinically, CT, MRI, and TransEsophageal Echocardiography (TEE) are reliable tools for diagnosing AD [4]. The doctor has the responsibility of screening for the patients with a high risk of AD to be further examined by CT, MRI or TEE. However, in initial diagnosis, the missed diagnosis rate of AD is high: 35.5% [5], 39.69% [6], 38.2% [7]; the missed diagnosis rate in acute aortic syndrome in the emergency room is even close to 80% [8]. Our CRST-Bagging model obtained a missed diagnosis rate of 19.2%, while SMOTEBagging’s missed diagnosis rate was 22.9% and RUSBoost’s missed diagnosis rate was 24.1%. Compared with clinical statistical data and other ensemble models, CRST-Bagging reduced the missed diagnosis rate of AD significantly. This means CRST-Bagging is an effective model to screen for AD patients in the early phase. It can help doctors make decisions about further correct treatment, especially in rural or remote areas.

Our study has certain limitations. First, our study is a retrospective analysis, and there were some missing data. We adopted the method of random filling by class, but this may lead to some bias. Second, due to the rarity of AD, our dataset includes only 802 AD patients of different ages. Our study took it as a whole without considering the difference in misdiagnosis rate and missed diagnosis rate of AD patients of different ages.

In the future, we will collect more patient data and divide the data into subgroups based on age. Studies will be performed in each data sub-group to reduce misdiagnosis and missed diagnoses. Based on the AD data, analytical results, and our proposed auxiliary diagnostic model, we will study the pathological mechanism and key diagnostic indicators of AD from the perspective of interpretability and explore whether there is a more definite clinical diagnostic method for AD.

## 6. Conclusions

In this paper, we proposed a cluster-based ensemble learning model named CRST-Bagging to assist in screening for AD by using routine examination data. In this model, we have proposed a cluster-based resampling method (CRST) to solve the problem of highly imbalanced data in the AD dataset. Then we used the Bagging algorithm to classify the AD and non-AD patients. We used the data from Xiangya hospital to evaluate the effectiveness of our resampling method and the prediction ability of our model. The experimental results show that our resampling method can effectively solve the problem of the imbalanced AD dataset. In addition, the performance of CRST-bagging is reasonably good at predicting AD patients, which can effectively decrease the number of missed diagnosis cases while ensuring a low number of misdiagnosis cases. Due to the rarity and complexity of AD, as well as the insufficiency of medical equipment in Chinese rural or remote areas, it is difficult for clinicians to diagnose AD, thus missing the best time for the patient’s treatment. Our model can help clinicians screen for AD patients in the early phase so that recommendations for further treatment can be made. This is an effective way to save AD patients’ lives, especially in areas without sufficient clinicians and medical equipment.

## Figures and Tables

**Table 1:** CRST Sampling Method.

**Input:** All standardized training sample set P; **Output:** The new balanced sub-dataset Z
Method: **1.** Divide the input training sample set P into the majority sample set $P_{\max}$ and the minority sample set $P_{\min}$ according to the labels, and then remove the labels; **2.** Use the K-means++ algorithm to cluster the training sample set $P_{\max}$ of majority classes to obtain K clusters. K is obtained by super-parameter optimization, denoted as: $\Omega =\left\{C_{1},C_{2},\cdots ,C_{K}\right\}$, wherein, $C_{i}=\left\{d_{i1},d_{i2},\cdots ,d_{\mathrm{im}}\right\}$ m represents the sample’s number of $C_{i}$; **3.** Take p% samples for each cluster class to obtain a new K cluster class sample set randomly, denoted as $\Omega^{\prime }=\left\{C_{1}^{\prime },C_{2}^{\prime },\cdots ,C_{K}^{\prime }\right\}$, wherein $C_{i}^{\prime }=\left\{d_{i1},d_{i2},\cdots ,d_{\mathrm{in}}\right\},n=m*p\%$ Combine the majority class sample set $\Omega^{\prime }$ and the minority class sample set $P_{\min}$ to synthesize the sample set Q. The S-T method is used for Q to obtain a balanced dataset Z.

**Table 2:** Experimental results for the XGBoost, Smote, S-T, CCST and CRST methods.

Method	Precision	Recall	F1
XGBoost	0.546	0.157	0.243
Smote	0.789	0.711	0.748
S-T	**0.793**	0.723	0.749
CCST	0.778	0.765	0.771
CRST	0.782	**0.774**	**0.778**

Note: The best scores are in bold.

**Table 3:** The experimental results of RUSBoost, SMOTEBagging, and CRST-Bagging on the original dataset using seven-fold cross-validation.

Method	Precision	Recall	F1	Training Time/Predicting Time (s)
RUSBoost	0.774	0.751	0.762	935.970/0.129
SMOTEBagging	0.791	0.780	0.785	98.458/0.133
CRST-Bagging	**0.841**	**0.783**	**0.811**	**49.695/0.069**

Note: The best scores are in bold.

**Table 4:** The experimental results of RUSBoost, SMOTEBagging and CRST-Bagging on the test set.

Method	Precision	Recall	F1	Predicting Time (s)
RUSBoost	0.788	0.759	0.773	0.036
SMOTEBagging	**0.842**	0.771	0.805	0.093
CRST-Bagging	0.838	**0.807**	**0.821**	**0.004**

Note: The best scores are in bold.

**Table 5:** Confusion matrix of RUSBoost (**a**), SMOTEBagging (**b**), CRST-Bagging (**c**) on the test set.

**(a) RUSBoost**		
	Confusion matrix on the test set	
	Predicted non-AD patient	Predicted AD patient
Actual non-AD patient	TN 135	FP 17
Actual AD patient	FN 20	TP 63
**(b) SMOTE Bagging**		
	Confusion matrix on the test set	
	Predicted non-AD patient	Predicted AD patient
Actual non-AD patient	**TN 140**	FP 12
Actual AD patient	FN 19	TP 64
**(c) CRST-Bagging**		
	Confusion matrix on the test set	
	Predicted non-AD patient	Predicted AD patient
Actual non-AD patient	TN 139	FP 13
Actual AD patient	FN 16	**TP 67**

Note: The best scores are in bold.

## Data Availability

A description of the dataset and features are provided in the manuscript. Since access to this dataset requires the approval of the hospital to which these EMRs belong, the dataset cannot be made publicly available.

## Supplementary Materials

The following supporting information can be downloaded at: https://github.com/wm-CSU/Aortic-dissection-dataset (accessed on 22 March 2022), Table S1: Description of Features.

## Abbreviations

**Abbreviation**	**Meaning**
AD	aortic dissection
LSTM	long short-term memory
PAVE	Pattern Attention model with Value Embedding
CT	Computed Tomography
AT: Ag	Antithrombin III antigen
PLGAg	Plasminogen antigen
S-TSH	Hypersensitivity thyrotropin
ESR	erythrocyte sedimentation rate
FT3	free triiodothyronine
XGBoost	eXtreme Gradient Boosting
T-SNE	t-distributed stochastic neighbor embedding
CRST	Cluster Random UnderSampling Smote–Tomek Approach
CRST-Bagging	Cluster Random Undersampling Smote–Tomek Bagging
S-T	Smote–Tomek
CCST	Cluster-Center Under-Sampling and Smote–Tomek Approach
